# How does perinatal maternal mental health explain early social inequalities in child behavioural and emotional problems? Findings from the Wirral Child Health and Development Study

**DOI:** 10.1371/journal.pone.0217342

**Published:** 2019-05-24

**Authors:** Callum Rutherford, Helen Sharp, Jonathan Hill, Andrew Pickles, David Taylor-Robinson

**Affiliations:** 1 Department of Public Health and Policy, Farr Institute, University of Liverpool, Liverpool, United Kingdom; 2 Department of Psychological Sciences, University of Liverpool, Liverpool, United Kingdom; 3 School of Psychology & Clinical Language Sciences, University of Reading, Reading, United Kingdom; 4 Biostatistics Department, Institute of Psychiatry, Psychology and Neuroscience, King’s College London, London, United Kingdom; University of North Carolina at Chapel Hill, UNITED STATES

## Abstract

**Background:**

This study aimed to assess how maternal mental health mediates the association between childhood socio-economic conditions at birth and subsequent child behavioural and emotional problem scores.

**Methods:**

Analysis of the Wirral Child Health and Development Study (WCHADS), a prospective epidemiological longitudinal study of the early origins of child mental health (n = 664). Household income at 20-weeks gestation, a measure of socio-economic conditions (SECs) in pregnancy, was the main exposure. The outcome measure was externalising and internalising problems, as measured by the Child Behaviour Checklist at 5 years. We assessed the association of household income with child behavioural outcomes in sequential linear models adjusting for maternal mental health in the pre- and post- natal period.

**Results:**

Children of mothers in more disadvantaged households had higher scores for externalising behaviour with a difference of 3.6 points comparing the most affluent to the most disadvantaged families (the socio-economic (SEC) gap). In our regression model adjusting for baseline confounders, comparing children of mothers in the most disadvantaged households to the least disadvantaged, we found that most disadvantaged children scored 45 percentage points (95% CI 9, 93) higher for externalising problems, and 42% of this difference was explained in the fully adjusted model. Adjusting for prenatal maternal depressive symptomology attenuated the SEC gap in externalising problems by about a third, rendering the association non-significant, whilst adjusting for pre- and post-natal maternal mental health attenuated the SEC gap by 42%. There was no significant relationship between household income and internalising problems.

**Conclusion:**

Social disadvantage is associated with higher child externalising behaviour problems score at age 5, and about 40% of this was explained by maternal perinatal mental health. Policies supporting maternal mental health in pregnancy are important to address the early emergence of inequalities in child mental health.

## Introduction

Reducing inequalities in mental health outcomes is a public health priority. [1] In the UK, one in ten children and young people (aged 5–16 years) have a clinically diagnosed mental health problem (behavioural 6% and emotional 4%). [2] The prevalence of mental health problems is a growing concern, with 24% of girls and 9% of boys aged 14 years self-reporting high levels of depression in the UK. [3] Poorer socioeconomic conditions (SECs) are associated with worse mental health outcomes. [4] A systematic review of 52 studies from 23 countries found that children and young people from disadvantaged families are two-to-three times more likely to develop mental health problems compared to economically advantaged children. [4] A number of theories describe the pathways through which SECs influence child health, including mental health, with the most commonly cited differentiating between material, psychosocial, behavioural and structural factors. [5] For example, experiences of poverty can have a negative impact on maternal mental health and behaviour, which in turn influences child health. [6]

Inequalities in mental health outcomes are established in the early years of a child’s development and these inequalities widen as children start primary education.[7] More support is needed earlier in the lifecourse to narrow inequalities. [7] In order to design and implement effective policies to tackle inequalities, a clearer understanding of the pathways, in the early years, is needed. Furthermore, it is unclear if these putative pathways differ for internalising problems (e.g., depression, anxiety) and externalising behaviour problems (e.g., aggression, attention) in children.

Maternal mental health is a well-established risk factor for child psychopathology and has been identified as a potential mediator of the association between socioeconomic conditions and child mental health outcomes. [8,9] One in five women experience depression in the perinatal period [10], although 60% of women are not detected and clinically diagnosed. [11] A previous episode of perinatal depression is associated with future risk [12], and around 15% of women who experience an episode of depression during pregnancy, experience a new episode within three months postpartum. [11]

Maternal mental health problems have been associated with the level of emotional support a child receives. Furthermore, poor parental mental health can affect children’s emotion regulation, development and attachment. [13,14] Few studies have explored the role of maternal mental health in generating social inequalities in child mental health. A greater understanding of the interplay and social patterning of maternal perinatal mental health and their impact on the socioeconomic gap in early child mental health problems is needed in order to design effective policy interventions to improve child mental health outcomes and reduce inequalities across the lifecourse.[15]

Using a prospective longitudinal study of child mental health trajectories, with frequent early-years measures, the aim of this study was therefore to assess the impact of childhood socio-economic conditions (SECs) on child internalising and externalising behaviours at age 5 years. We further aimed to explore the role of maternal mental health in explaining any social patterning of early child behavioural and emotional problems identified.

## Materials and methods

### Design, setting and data source

We analysed data from a longitudinal cohort study of children born on the Wirral, in the North West of England. The study recruited 1233 participants consecutively from first-time mothers who booked antenatal care between February 2007 and October 2008 at the Wirral University Teaching Hospital, North West of England. [16] Owing to the socio-demographic composition of the Wirral, 41.7% of the sample were in the most deprived quintile of deprivation at recruitment, using the English Index of Multiple Deprivation (IMD [17]. These indices combine economic, social and housing indicators measures at the census into a composite deprivation score for small areas in England.

WCHADS utilises multiple informant questionnaires alongside novel experimental and observational measures, embedded within a longitudinal design, to measure child social, emotional and behavioural functioning. [16] More information on the sampling design and cohort measures can be found online [https://www.liverpool.ac.uk/psychology-health-and-society/departments/psychological-sciences/research/first-steps/for-researchers/]. This study uses data on 759 children with data available on the CBCL at age 5 years included in the study (62% of the total sample), 664 of these (87.5%) had data on all covariates. A flowchart of the analysis sample is available in the supplementary materials (S1 File). The study was granted ethical approval by the Cheshire North and West Research Ethics Committee [REF: 05/Q1506/107 (June 2006); 10/H1010/4 (June 2010)]. This analysis did not require further ethical approval.

### Outcome measures

The main outcome measures were internalising and externalising problem subscale scores on the Child Behaviour Checklist (CBCL) at age five. When the cohort child was aged 4.5–5 years (58.5 months (3.6)) parents completed the preschool version of the Child Behaviour Checklist, a validated instrument, with high rates of validity and reliability across many populations, measuring child emotional and behavioural disorders. [18,19] The measure contains 99 problem items, scored 0 (not true), 1 (somewhat or sometimes true) and 2 (very true or often true). Internalising problems are derived from combining the anxious/depressed, emotionally reactive, withdrawn and somatic complaints scores, and externalising problems by combining aggressive behaviour and attention problem scores. [18] The subsequent main analysis uses raw scores (unadjusted for sex and age) for both internalising and externalising domains, treated as a continuous variable.

### Early-life risk factors

The primary exposure measure of childhood socio-economic conditions in pregnancy, our baseline, was household income measured at 20-weeks gestation. Mothers were asked ‘What is your approximate annual family income?’ with responses collected in £10,000 incremental bands (Range: Up to £10,000 –Over £71,000). Our analysis uses the socioeconomic gap, which is the difference between the least (Over £71,000, n = 53)) and most deprived (Up to £10,000, n = 48)) households, to explore inequalities in mental health outcomes. In sensitivity analyses, we used IMD quintile score as an alternative exposure of childhood socio-economic conditions.

For maternal mental health, we used total score on the Edinburgh Postnatal Depression Scale (EPDS). The EPDS is a self-reported 10-item measure used as screening tool for depression in the perinatal period. [20] We used the EPDS as a continuous measure, [20] measured at 20-weeks gestation, 14 months postnatal and 3 ½ years as a proxy for maternal mental health. Although primarily used a measure for depressive symptoms, the EPDS has been shown to identify anxiety and has a potential use as a screening tool for other mental disorders. [21]

Demographic characteristics that are potentially associated with child mental health outcomes included as baseline variables in the analysis: maternal age at consent coded as 18–24, 25–34, 35+), child’s age, sex and ethnicity. As only 2.9% of the sample identified as other than White-British, we dichotomised ethnicity to be ‘White-British’ or ‘Other’.

### Analysis strategy

First, we undertook a univariable analysis estimating association between covariates of interest and total CBCL internalising and externalising problem scores, using linear regression. Due to the outcome data being highly positively skewed, the total scores were log-transformed, with exponentiated beta coefficients interpreted as a geometric means, whereby, a change in the independent variable is represented as a ratio of change in the dependent variable. [22]

A hierarchical approach was used for the multivariate regression modelling. Firstly, we fitted baseline models for each of our two outcomes by family income, treated as a linear function. Secondly, we adjusted for baseline demographic variables: maternal age at recruitment to the study, sex and ethnicity. We then fitted models with measures of maternal depression at our three separate time periods added separately (antenatally; at 14 months; and 3.5 years); and finally, we adjusted for maternal mental health at all three time points.

**Fig 1:**
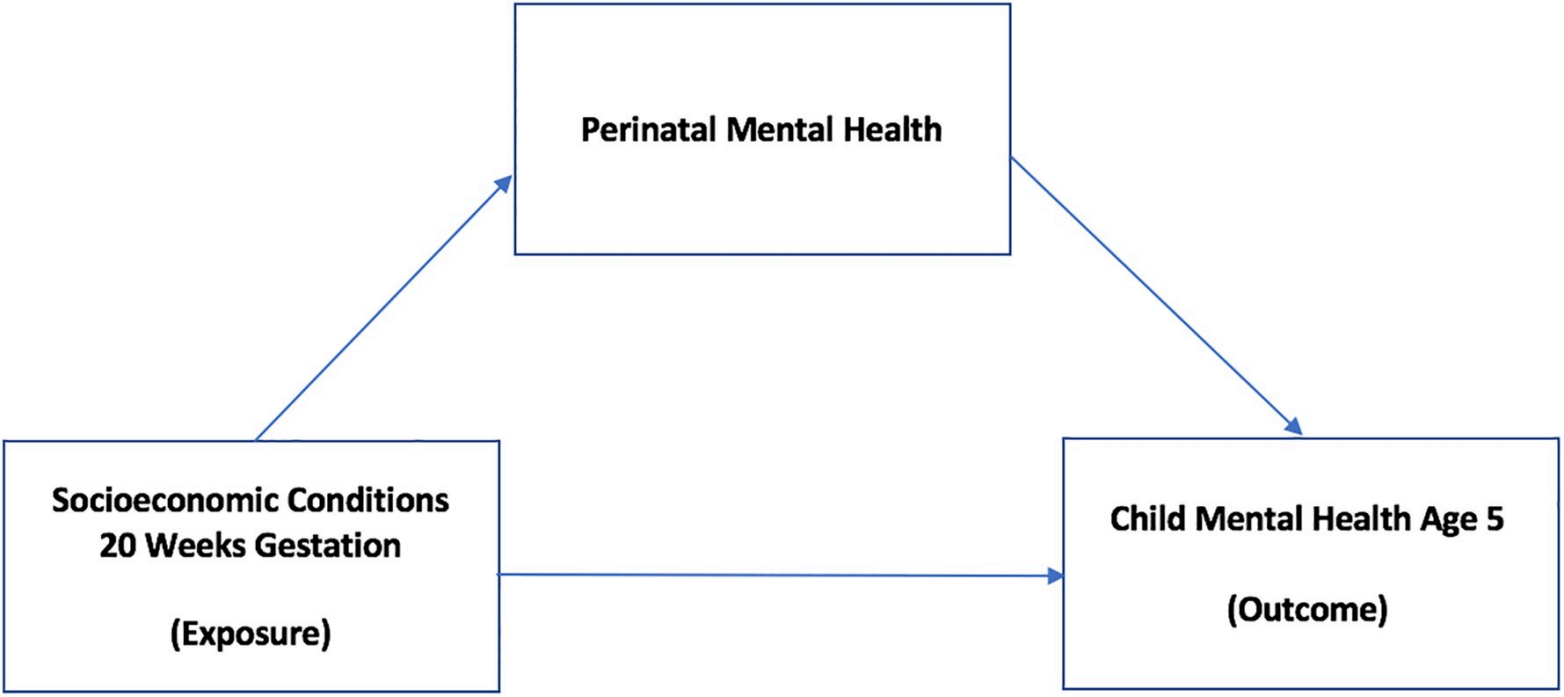
Pathway diagram showing the potential association between socioeconomic conditions (SECs) and child behavioural and emotional problems, with maternal mental health as a potential mediator.

We took any attenuation in the geometric mean income coefficient on the addition of measures of maternal mental health in a complete case sample to indicate potential mediation of any socio-economic gap in mental health outcomes, [23,24] calculated as 100x(coefficient-adjusted coefficient)/(coefficient -1) to estimate the change in relative gap in behavioural and emotional problems on the basis of family income. [25] (Fig 1).

The socioeconomic gap measures health inequalities between the most and least deprived groups. It is an important tool as often population averages overlook differences in health between groups. This approach has been used in previous studies of health inequalities in social epidemiology. [23,24]

Missing data was handled using listwise deletion. To account for item missingness on the Edinburgh Postnatal Depression scale we used mean substitution. [26] Analyses were conducted using R software (version X).

### Robustness test

We undertook a number of sensitivity analyses to test our assumptions. We repeated our analysis using the index of multiple deprivation (IMD) quintile as an alternative measure of socioeconomic conditions [27]. We repeated our analysis using clinical cut-offs for internalising and externalising behaviour problems (0 = no clinical level score, 1 = borderline/clinical level score) to assess the association between SEC and clinical levels of child behavioural and emotional problems. Sensitivity analyses were performed using multiple imputation by chained equations to impute missing data values for all of the variables included in the models. We also undertook a formal mediation analysis using the counterfactual framework to assess how much of the effect of SEC (household income) on child behavioural problems is mediated via maternal depressive symptomology measured at all three time points. [S1 File]

## Results

Table 1 shows sample characteristics stratified by household income. There were differences in mean scores for externalising and internalising behaviour problems by household income. As household income increases, mean scores for internalising and externalising behaviour problems decrease. Scores ranged from 9.9 (8.1 SD) in the lowest income households to 6.3 (6.3 SD) in the highest income households for externalising problems and from 8.1 (5.6 SD) to 5 (4.2 SD) for internalising problems. For all measures of maternal depressive symptoms in the perinatal period, mothers from the lowest income households had higher mean scores. Higher maternal age (> 35 years) was more common in the higher household income groups.

**Table 1:** Characteristics of the study population by household income.

	Total	Up to £10,000	£10–20,000	£21–30,000	£31–40,000	£41–50,000	£51–60,000	£61–70,000	Over £71,000	p-value#
**Subjects n (%)**	666 (100)	48 (7.2)	77(11.6)	97(14.6)	134(20.1)	110(16.5)	101(15.2)	46(6.9)	53(8.0)	
**Externalising raw score**	7.7 (6.8)	9.9 (8.1)	10.9 (8.9)	7 (6.5)	6 (5.4)	7.8 (6.7)	7.5 (6.4)	7.6 (5.3)	6.3 (6.3)	< 0.001
**Internalising raw score**	6.2 (5.5)	8.1 (5.6)	7.9 (6.7)	5.5 (5.8)	5.1 (4.9)	6.5 (4.9)	6.7 (5.7)	5.6 (5)	5 (4.2)	< 0.001
**Log-Externalising raw score**	1.8 (0.9)	2.1 (0.9)	2.2 (0.9)	1.7 (0.9)	1.6 (0.9)	1.8 (1)	1.8 (0.9)	2 (0.7)	1.6 (0.9)	< 0.001
**Log-Internalising raw score**	1.7 (0.8)	2 (0.7)	1.9 (0.8)	1.5 (0.9)	1.5 (0.8)	1.8 (0.8)	1.8 (0.7)	1.6 (0.8)	1.5 (0.8)	< 0.001
**Externalising clinical score (%)**	29 (4.4)	3 (6.3)	10 (13.0)	3 (3.1)	1 (0.8)	5 (4.5)	5 (5.0)	1 (2.2)	1 (1.9)	< 0.01
**Internalising clinical score (%)**	58 (8.7)	6 (12.5)	12 (15.6)	7 (7.2)	9 (6.8)	10 (9.1)	9 (8.9)	4 (8.7)	1 (1.9)	0.222
**Maternal age years n (%)**										
18–24	160 (24.1)	40 (83.3)	44 (57.1)	36 (37.1)	30 (22.6)	6 (5.5)	2 (2.0)	2 (4.3)	0 (0)	< 0.001
25–34	408 (61.4)	5 (10.4)	29 (37.7)	50 (51.5)	83 (62.4)	94 (85.5)	78 (78.0)	33 (71.7)	36 (67.9))	
35+	96 (14.5)	3 (6.3)	4 (5.2)	11 (11.3)	20 (15.0)	10 (9.0)	20 (20.0)	11 (24.0)	17 (32.1)	
**Child age (months)**	58.5 (3.6)	58.2 (4.5)	58.4 (3.9)	58.4 (3.1)	58.7 (4.2)	58.5 (3.3)	58.8 (3.3)	58.1 (2.6)	58.5 (3.6)	0.965
**Child’s sex: female (%)**	349 (52.4)	28 (58.3)	47 (61.0)	54 (55.7)	66 (49.3)	53 (48.2)	54 (53.5)	15 (32.6)	32 (60.4)	0.064
**Child’s ethnicity: other (%)**	19 (2.9)	1 (2.1)	3 (3.9)	3 (3.1)	4 (3.0)	4 (3.6)	2 (2.0)	1 (2.2)	1 (1.9)	0.996
**Maternal depression**										
20-weeks gestation	6.4 (4)	9.2 (4.8)	8.1 (4.2)	7.1 (4.4)	5.7 (3.5)	6.2 (3.7)	5.5 (3.8)	4.5 (3.2)	5.2 (3.2)	< 0.001
14 months	5.1 (4)	6.9 (5.1)	6 (4.4)	5.9 (4.5)	4.8 (3.6)	4.7 (3.3)	4.6 (3.8)	4.1 (3.2)	3.9 (3.1)	< 0.001
3.5 years	5 (3.9)	7.5 (6)	5.6 (4.4)	5.6 (3.7)	4.7 (3.7)	4.6 (3.4)	4.4 (3.2)	4.3 (3)	4.4 (3.3)	< 0.001

Data are presented as means (SD), unless otherwise stated. Item missingness: Maternal age 2

^#^ F-test: externalising, internalising, maternal depression; Chi-squared: Maternal age, Child’s sex; Fisher’s Exact: Ethnicity

### Associations between covariates and child behavioural and emotional problems

Lower household income was associated with higher scores for externalising problems (Table 2) but there was no significant association with internalising problems. Boys had significantly higher scores for externalising problems, whilst there were no significant sex differences for internalising problems. Consequently, the multivariable analysis focuses on externalising problems. In the univariable analysis male sex, child’s age and maternal depressive symptoms at 20-weeks gestation, 14 months and 3 ½ years postnatally were associated with increased externalising mental health scores. By contrast there was no association with maternal age and ethnicity. To address the assumptions of mediation, we established that household income was associated with increased scores on the EPDS, using linear regression. (20 weeks (ß 0.58 p <0.001); 14 months (ß 0.40, p <0.001) and at 3.5 years (ß 0.37, p<0.001)).

**Table 2:** Univariable associations (n = 666).

		Externalising Raw Scores				Internalising Raw Scores		
Variables (Reference group)	Exp(ß)	95% LCI	95% UCI	p-value	Exp(ß)	95% LCI	95% UCI	p-value
**Primary Exposure**								
Household Income	1.04	1.01	1.08	<0.05	1.03	1.00	1.06	0.068
SEC Gap*	1.32	1.04	1.69	<0.05	1.23	0.98	1.53	0.068
**Socio-demographic**								
Mat Age (ref: 35+)				0.642				0.614
18–24	1.11	0.88	1.39		1.10	0.89	1.35	
25–34	1.09	0.90	1.33		1.09	0.91	1.30	
Child Age (Months)	0.97	0.95	0.99	<0.01	0.97	0.95	0.99	<0.001
Sex (Male)	1.22	1.07	1.40	<0.01	1.05	0.92	1.18	0.473
Ethnicity (Other)	0.79	0.53	1.19	0.265	1.25	0.86	1.81	0.234
**Maternal Depression**								
20-weeks gestation	1.04	1.07	1.40	<0.001	1.05	1.03	1.06	<0.001
14 months postnatal	1.03	1.02	1.05	<0.001	1.04	1.02	1.06	<0.001
3.5 years postnatal	1.04	1.02	1.06	<0.001	1.06	1.04	1.08	<0.001

Note: Exp (ß) interpreted as geometric means. Maternal Age (Ref: 35+); Sex (Ref: female); Ethnicity (Ref: white British).

*The socioeconomic (SEC) gap, the difference between the most and least deprived households.

### Association between exposure and outcome, adjusted for other early-life factors

Table 3 shows the results of the multivariable analysis for externalising problems (results for internalising problems are available as supplementary material (S1 File). In the unadjusted model (model 1), children in the most deprived households had scores 32% (95% CI 4, 69) higher for externalising problems, compared to children from the least deprived households. Adjusting for socio-demographic factors slightly increased the household income coefficient such that children in the most disadvantaged households had scores 45% (95% CI 9, 93) higher than those in the least disadvantaged.

**Table 3:** Multivariable analysis. Child Mental Health Life Course Models (n = 664).

	Model I	Model II	Model III	Model IV	Model V	Model VI
	Exp (ß)	Exp (ß)	Exp (ß)	Exp (ß)	Exp (ß)	Exp (ß)
CBCL Externalising Raw Scores						
Household Income	1.04*	1.06*	1.04	1.04*	1.04*	1.03
	(1.01, 1.08)	(1.01, 1.10)	(0.99, 1.08)	(1.00, 1.09)	(1.00, 1.09)	(0.99, 1.80)
Household Income (SEC Gap)	1.32**	1.45**	1.31	1.34*	1.34*	1.26
	(1.04, 1.69)	(1.09, 1.93)	(0.98, 1.75)	(1.00, 1.79)	(1.00, 1.77)	(0.95, 1.69)
Child Age		0.97**	0.97**	0.97**	0.97**	0.97**
		(0.95, 0.99)	(0.96, 0.99)	(0.95, 0.99)	(0.95, 0.99)	(0.96, 0.99)
Maternal Age (18–24)		0.95	0.92	0.96	0.94	0.93
		(0.74, 1.22)	(0.72, 1.18)	(0.75, 1.23)	(0.74, 1.21)	(0.73, 1.19)
Maternal Age (25–34)		1.05	1.04	1.06	1.05	1.05
		(0.86, 1.28)	(0.85, 1.26)	(0.87, 1.29)	(0.87, 1.28)	(0.87, 1.28)
Sex (Male)		1.24**	1.23***	1.22**	1.24**	1.22**
		(1.08, 1.42)	(1.08, 1.40)	(1.07, 1.40)	(1.08, 1.41)	(1.07, 1.40)
Ethnicity (Other)		0.80	0.80	0.82	0.85	0.84
		(0.53, 1.21)	(0.53, 1.21)	(0.54, 1.23)	(0.56, 1.28)	(0.56, 1.26)
Maternal Prenatal Depression			1.03****			1.02
			(1.01, 1.05)			(0.99, 1.04)
Maternal Postnatal Depression (14 months)				1.03***		1.01
				(1.01, 1.05)		(0.99, 1.03)
Maternal Postnatal Depression (3.5 Years)					1.04***	1.02*
					(1.02, 1.06)	(1.00, 1.05)

Note: Exp (ß) interpreted as geometric means. Maternal Age (Ref: 35+); Sex (Ref: female); Ethnicity (Ref: white British)

*p<0.05;

**p<0.01;

***p<0.001.

The socioeconomic (SEC) gap, the difference between the most and least deprived households.

**Fig 2:**
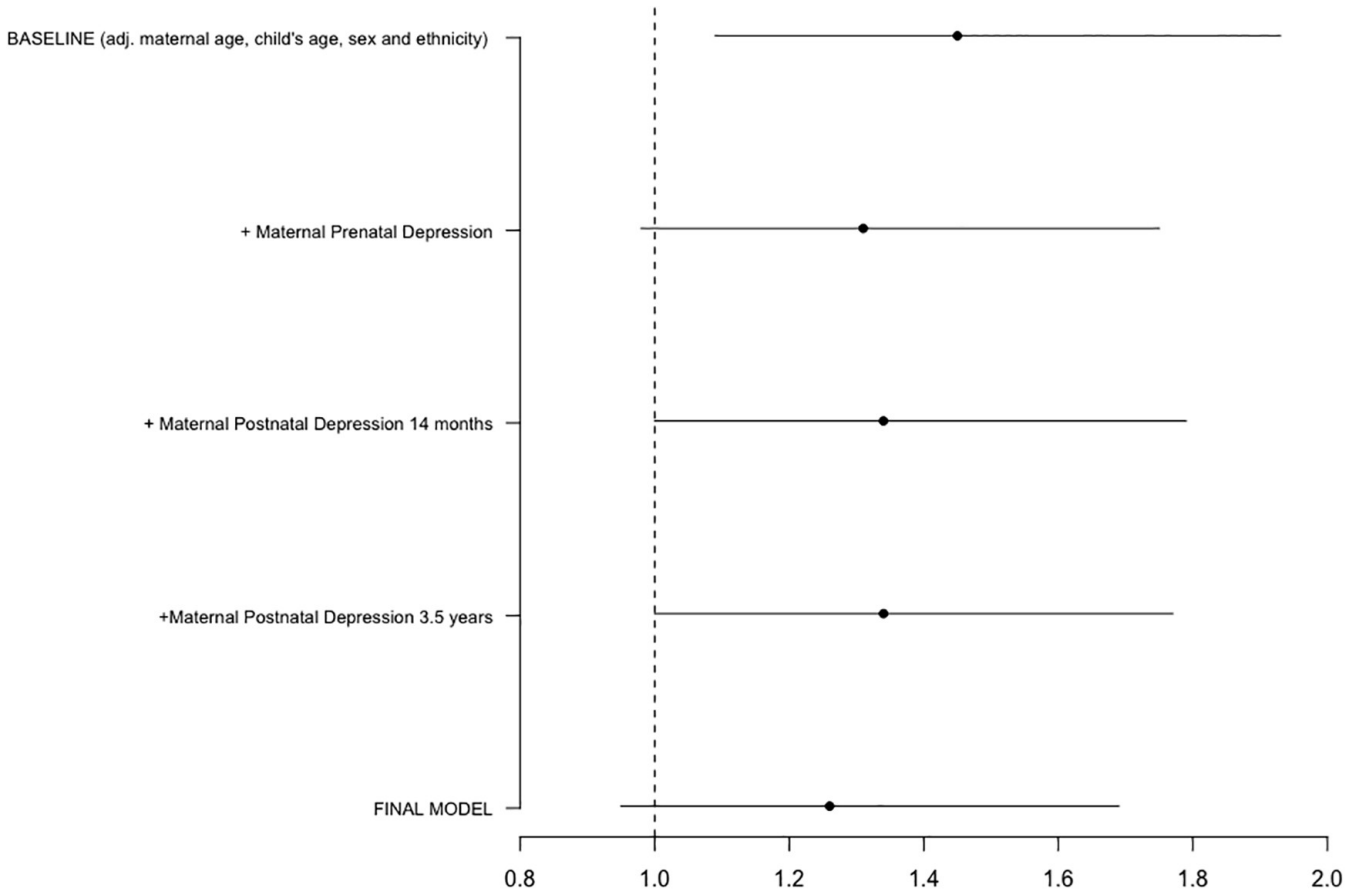
Attenuation of the socioeconomic gap in child externalising behaviour problems independently adjusting for maternal depressive symptoms.

Fig 2 shows how independently adjusting for maternal depressive symptoms across the perinatal period attenuates the socioeconomic gap in child externalising behaviour problems. Adjusting for maternal prenatal depressive symptoms antenatally (model 3) the income gap coefficient is attenuated by 31% (Exp(ß**)** 1.31 [95% CI 0.98, 1.75]), from baseline (model 2), rendering the association with household income non-significant. Adjusting for either maternal depressive symptoms at 14 months or 3 ½ years postnatally (models 4 & 5) attenuated the socioeconomic gap in child externalising problems by 24% (Exp(ß**)** 1.34 [95% CI 1.00, 1.79]), from baseline, and the association with household income remained significant in both models. Finally, adjusting for maternal depressive symptoms at all three time points (model 6) attenuated the socioeconomic gap to non-significance, attenuating the SEC gap by 42% (Exp(ß**)** 1.26 [95% CI 0.95, 1.69]) compared to model 2.

In the sequential models maternal depressive symptoms entered at any timepoint (prenatal, 14 months and 3.5 years) was associated with an increased score for externalising problems. A one unit increase in EPDS score increased externalising scores by 3% (95% CI 1, 5) in the prenatal period and by 4% (95% CI 2, 6) in the postnatal period. Comparing the effect of depressive symptoms between mothers from the most deprived households to the least deprived (calculated as Exp(ß x difference in mean scores)), depressive symptoms in the prenatal period increased externalising scores by 13% (95% CI 6, 22) and by 9% (95% CI 3, 15) and 12% (95% CI 6, 18) for 14 months and 3.5 years postnatally. In our fully adjusted model (model 6), only maternal depressive symptoms at 3 ½ years postnatally, child’s age and sex remained significant. Male sex increased scores for externalising problems by 22% (95% CI 7, 40).

### Robustness tests

In this study we identified a gap in average CBCL scores across the socioeconomic spectrum of about 3 CBCL points at age 5 years, or one third of a z-score using the untransformed CBCL scores. To contextualise this difference, the effect size for the SEC gap on externalising behaviour problems is similar to the effect size of an evidence-based parenting intervention in a recent meta-analysis and larger than the gender/sex effect in our analysis. [28]

Furthermore, we repeated our analysis using clinical cut-offs for internalising and externalising behaviour problems (0 = no clinical level score, 1 = borderline/clinical level score) to assess the association between SEC and clinical levels of child behavioural and emotional problems. This showed a social gradient in the proportion of children with behaviour problems (Table 1), and our regression modelling using these dichotomised outcomes showed similar results to our main analysis using average values. In the age and sex baseline adjusted model there was an 18% increased risk of behaviour problems across the socio-economic gap. (S1 File).

Using IMD as an alternative measure of SECs, children in the most deprived quintile scored 39% higher (95% CI 8, 80) for externalising problems compared to the least deprived children. Adjusting for maternal depressive symptoms across the perinatal period slightly attenuated the effect to 35% (95% CI 4, 73) in the final model, a reduction of 11%. Male sex and maternal depressive symptoms at all time points significantly increased externalising problem scores (S1 File). We found similar results using a counterfactual framework approach to mediation compared with the approach used in the main analysis (S1 File).

## Discussion

### Main findings

Using a prospective longitudinal study design with frequent early years measures of child and maternal mental health we show that low SECs in childhood, measured on the basis of household income at 20-weeks gestation, is associated with higher scores for externalising behaviour problems in children by age 5 years, but not for internalising problems. Children growing up in the most disadvantaged circumstances scored about 45% higher for externalising problems compared to children growing up in the least disadvantaged circumstances, with boys scoring significantly higher. About 40% of this association was explained by the increased prevalence of maternal mental health problems, particularly pre-natal mental health, in mothers of children growing up in disadvantaged circumstances. Our analysis suggests that interventions that improve perinatal maternal mental health are likely to reduce social inequalities in child externalising behaviour problems.

### Comparison with other findings

Our results corroborate previous studies suggesting that exposure to disadvantaged socioeconomic conditions increases the risk of later child mental health problems. [4,29–32] Socio-economic disadvantage in the early years also has profound effects throughout the lifecourse. Longitudinal studies have shown that the SEC gap in child behavioural and emotional problems can be observed from infancy and that inequalities are maintained or increased throughout early childhood into adolescence. [33,34]

Furthermore our results concur with studies that have shown that the relationship between SECs and behaviour problems is stronger for externalising symptoms. [30,35,36] Whilst our study only found a significant relationship between household income and externalising problems, the relationship with internalising problems did not quite reach significance, and this may be due to our relatively small sample size and sample age. However, this finding should not be overlooked, potential mechanisms of inequalities in internalising problems may differ compared to externalising problems and manifest later in the lifecourse.

The development and prevalence of internalising problems has been associated with increasing age. [37] Although associations between SECs and internalising problems in children have been demonstrated in a number of other larger studies across childhood [29,38,39]. Furthermore, our study found that child’s age at time of CBCL assessment significantly decreases externalising problem scores (Exp(ß**)** 0.97 [95% CI 0.96, 0.99]). Studies have shown that the prevalence of externalising problems decline with age and this occurs across socioeconomic groups. [33,40]

Maternal psychopathology has been identified as risk factor that mediates the association between socioeconomic status and child and adolescent mental health problems at later ages [4,8,41], with effects on child mental health well documented. [9] Kiernan et al [39] found that maternal depression mediated ~30% of the total economic effect on externalising problems, a similar figure to that found when we adjusted for postnatal measures of maternal depression in our study. Our study extends the evidence base further, by showing how maternal mental health in the perinatal period impacts on the socioeconomic gap in very early measures of child behaviour problems at age 5 years. We show that low income is associated with higher scores of maternal depressive symptoms, and this is in turn related to higher externalising problem scores for children growing up in more disadvantaged SECs. Whilst we found that perinatal mental health is an important mediator of inequalities in child externalising behaviour problems, further studies should explore the mediating role of other perinatal factors such as birthweight and breastfeeding.

Our study demonstrated clear sex differences, with boys at greater risk for externalising problems at age five, after adjustment for a range of covariates in our final model, corroborating previous studies. [8,9,42] There was no association between sex and internalising problems. Research suggests that the pathways and mechanisms for internalising and externalising problems may differ for boys and girls throughout development. Boys may be more susceptible to the impact of adverse environmental stresses, or their behaviour may be met by differing patterns of maternal response. [43]

### Policy and practice implications

In the UK, the prevalence of child and adolescent mental health problems is increasing. [3,44] This study has shown that there are very early social inequalities in child behavioural problems. It is essential that policies to address inequalities focus on improving social conditions for all children, whilst also acting on maternal mental health in the perinatal period can potentially reduce the SEC gap in preschool-aged children’s externalising behaviour problems.

This study also highlights the importance of maternal mental health in perinatal period for children across the lifecourse. In the UK, maternal mental health problems in the perinatal period have been estimated to cost society £8.1 billion for each one-year cohort of births, with ~ 75% of the cost relating to impacts on the child. [45] Risk starts before birth and estimates suggest that approximately 122,000 babies (under 1) in the UK live with a parent with mental health problems. [1]

### Strengths and limitations

This study used data from a prospective longitudinal study with multiple measures of maternal mental health in the perinatal period. WCHADS is a rich dataset, with socio-demographic and environmental risk factors collected during pregnancy and the early-years. A limitation of this study is that our measure of child mental health is based upon maternal report and not a clinical diagnosis. However, we utilised the CBCL 1.5–5, a well-established and validated tool for screening at risk, preschool aged, children that can be used in clinical settings. [46] Our aim was to explore the effects of socio-economic conditions on broad child behavioural and emotional problems and therefore treated CBCL scores as continuous. Although clinical cut offs are available, a dimensional approach better captures the development of behavioural problems, as sub-clinical difficulties are associated with potential clinical disorders over the lifecourse. [47] Furthermore, repeating the analysis using clinical cut-offs led to similar conclusions.

Additionally, maternal mental health at the time of CBCL measurement can influence responses. We have not controlled for rater-bias in the main analysis since at age 5 years, as maternal mental health at age 5 is likely to be on the causal pathway between SECs and child behaviour and emotional problems. Controlling for maternal depressive symptoms at the time of the outcome measure is likely to be an over-adjustment. However, in sensitivity analysis we controlled for maternal depressive symptomology at this timepoint using the Centre for Epidemiological Studies Depression scale (CES-D) and the results are similar (S1 File).

Another limitation of this study is that our measure of maternal mental health relies on a self-report and not a clinical diagnosis. Although, the EPDS is a validated measure of depressive symptomology in the perinatal period its use at 3.5 years postnatal may not fully capture the severity of depressive symptoms later in the lifecourse.

WCHADS retains a large sample size for a developmental study which allows for rich data to be collected on socio-demographic and child behavioural and emotional problems in the perinatal period and offers the potential to investigate causal pathways in the early years. However, as a consequence of cohort attrition our study used data available on 62% of children from the original sample population. Despite this, the socio-demographic composition of WCHADS participants permits analysis exploring the effect of SECs. Although, caution is needed as our measure of SECs relies on self-reported income and does not account for family size or resource allocation. There may not be sufficient statistical power to identify small effect estimates in our study, and effects may not necessarily generalisable to the UK population, though they are likely to be generalisable to similarly disadvantaged areas.

Whilst we have conducted a counterfactual mediation (S1 File) to understand the potential causal pathway using variables that are temporally ordered, further research should explore the complex bi-directional relationship between family SECs and maternal and child behavioural and emotional problems over time. Reassuringly, our sensitivity analysis using different outcome and exposure measures produced similar results and conclusions (S1 File).

## Conclusion

More knowledge is needed for the earliest possible identification of children with behavioural and emotional problems. Whilst we have shown that income and deprivation impact maternal mental health and child externalising behaviour problems, there may be threshold effects, and identifying these in future research may usefully inform public health and welfare policy. Further longitudinal investigation, with an early-years focus, is welcomed.

Currently, around 70% of children and adolescents with mental health problems have not had appropriate early intervention. [48] Efforts to reduce inequalities in child behavioural and emotional problems should focus on reducing socioeconomic inequalities, with multi-dimensional strategies and prevention across the lifecourse. Our analysis suggests targeting maternal mental health in the perinatal period is important, but that this alone will not address the increased risk in disadvantaged children.

## Supporting information

S1 File Supplementary materials. (DOCX)
