# Supplementary material for: How does perinatal maternal mental health explain early social inequalities in child behavioural and emotional problems? Findings from the Wirral Child Health and Development Study
Source: PLoS One. 2019 May 24;14(5):e0217342. doi: 10.1371/journal.pone.0217342 (PMC6534344; doi:10.1371/journal.pone.0217342)
Supplement: S1 File — (DOCX) [file pone.0217342.s001.docx]

**Assessing the impact of perinatal maternal mental health on early inequalities in child mental health**

C Rutherford^1^, H Sharp^2^, J Hill^3^, A Pickles^4^, D Taylor-Robinson^1^

Online Supplementary Materials

**S1: Mediation analysis using counterfactual framework to assess the proportion of the effect of income on child mental health mediated through maternal mental health**

We undertook a formal mediation analysis using the counterfactual framework to assess how much of the effect of SEC income on child mental health is mediated via maternal mental health measured at all three time points.

The figure below shows the directed acyclic graph (DAG) for a causal mediation analysis using the counterfactual framework. We estimated the Natural Direct Effect (NDE), Natural Indirect Effect (NIE) and Total Effect (TE) for the the directed acyclic graph (DAG) in figure S1, adjusting for maternal age at recruitment, ethnicity, child sex and maternal pre- and post-natal mental health, using the *medflex* package in R software. This package offers a flexible set of ready-made functions for fitting natural effect models, which is a novel class of causal models to directly parameterize the path-specific effects of interest, and can accommodate multiple correlated mediators (Steen et al., 2017). We calculated the proportion mediated via maternal mental health applying the formula NIE/(NDE+NIE) (Vanderweele, 2015).

Overall 37% of the total effect of socio-economic conditions (low versus high) on child mental health is mediated through maternal mental health (figure S1), with a total effect of 1.055 (95% CI 1.013 to 1.098) and NIE of 1.020 (95%CI 1.009 to 1.031).

For the mediation analysis to have a causal interpretation, we assume no exposure/mediator interaction; that adjustment for the four types of confounding has been addressed and that there is no post-treatment confounding. The four types of confounding are: (1) confounding of the exposure-outcome relationship; (2) confounding of the mediator-outcome relationship; (3) confounding of the exposure-mediator association; and (4) mediator-outcome confounders also affected by the exposure. (Vanderweele, 2015).

**Figure S1: Causal mediation analysis DAG**


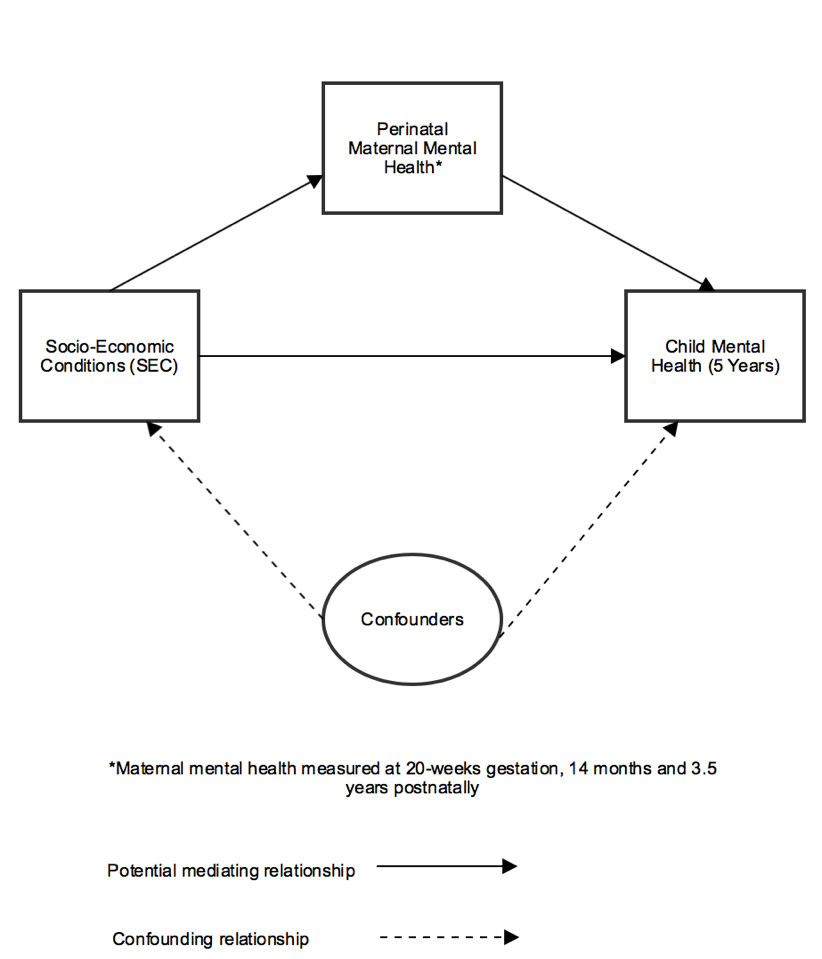


**Mediation Analysis Results**

| **Mediation Analysis** |  |  |  |  |  |  |
| --- | --- | --- | --- | --- | --- | --- |
| **Effect Breakdown** | **(ß)** | **95% LCL** | **95% UCL** | **Exp (ß)** | **95% LCL** | **95% UCL** |
| Natural Direct Effect | 0.033 | -0.006 | 0.073 | 1.034 | 0.994 | 1.076 |
| Natural Indirect Effect | 0.020 | 0.009 | 0.031 | 1.020 | 1.009 | 1.031 |
| Total Effect | 0.053 | 0.013 | 0.093 | 1.055 | 1.013 | 1.098 |
| Exposure: household Income. Confounders: maternal age, ethnicity, child sex, maternal mental health | | | | | |  |
| (20 weeks gestation, 14 months & 3.5 years postnatal) | | |  |  |  |  |

1. Vanderweele, T.J. Explanation in Causal Inference: Methods for Mediation and Interaction. Oxford; 2015.
2. Steen J, Loeys T, Moerkerke B, Vansteelandt S. Medflex: an R package for flexible mediation analysis using natural effect models. *Journal of Statistical Software*. 2017;76(11).

**S2: Internalising and Externalising Life Course Models Controlling for Rater Bias.**

We ran lifecourse models controlling for rater-bias at Child Behaviour Checklist (CBCL) measurement at 5 years. This was not included in the main analysis due to the change of measurement for maternal mental health. We used the Edinburgh Postnatal Depression Scale (EPDS) as a proxy for maternal mental health in the perinatal period, however, this measure was not available for our analysis sample at age 5 years. [1] To control for rater bias we used the Centre for Epidemiological Studies-Depression scale (CES-D), a 20-item measures that captures reporter experience of depression symptoms. [2] For this analysis we used total score on the CES-D, as a continuous measure. The results are similar to our main analysis, showing that after adjusting for maternal mental health at age 5 years, the association between household income and externalising behaviours becomes non-significant.

[1] Cox JL, Holden JM, Sagovsky R. Detection of postnatal depression: Development of the 10-item Edinburgh Postnatal Depression Scale. *British Journal of Psychiatry* 1987;150:782–6. doi:10.1192/bjp.150.6.782

[2] Radloff, L. S. The CES-D scale: A self-report depression scale for research in the general population. *Applied Psychological Measurements* 1977; 1:385-401.

| **Child Mental Health Life Course Models (n=663)** | | | | | | |
| --- | --- | --- | --- | --- | --- | --- |
|  | Model I | Model II | Model III | Model IV | Model V | Model VI |
|  | Exp (ß) | Exp (ß) | Exp (ß) | Exp (ß) | Exp (ß) | Exp (ß) |
| **CBCL Externalising Raw Scores** |  |  |  |  |  |  |
| Household Income | 1.026 | 1.041 | 1.033 | 1.037 | 1.037 | 1.031 |
|  | (0.991, 1.062) | (0.999, 1.084) | (0.991, 1.076) | (0.996, 1.080) | (0.996, 1.080) | (0.990, 1.075) |
| Child Age |  | 0.975^**^ | 0.976^*^ | 0.975^**^ | 0.975^**^ | 0.975^**^ |
|  |  | (0.957, 0.993) | (0.958, 0.994) | (0.957, 0.993) | (0.957, 0.993) | (0.958, 0.994) |
| Maternal Age (18-24) |  | 0.959 | 0.933 | 0.962 | 0.950 | 0.935 |
|  |  | (0.751, 1.225) | (0.730, 1.192) | (0.753, 1.229) | (0.744, 1.213) | (0.731, 1.195) |
| Maternal Age (25-34) |  | 1.058 | 1.048 | 1.062 | 1.057 | 1.051 |
|  |  | (0.872, 1.283) | (0.865, 1.271) | (0.875, 1.288) | (0.872, 1.282) | (0.867, 1.275) |
| Sex (Male) |  | 1.214^**^ | 1.210^**^ | 1.211^**^ | 1.218^**^ | 1.213^**^ |
|  |  | (1.063, 1.387) | (1.060, 1.381) | (1.060, 1.382) | (1.067, 1.390) | (1.063, 1.385) |
| Ethnicity (Other) |  | 0.838 | 0.832 | 0.841 | 0.857 | 0.846 |
|  |  | (0.558, 1.258) | (0.555, 1.247) | (0.560, 1.262) | (0.571, 1.286) | (0.564, 1.270) |
| Maternal Prenatal Depression |  |  | 1.022^*^ |  |  | 1.016 |
|  |  |  | (1.004, 1.040) |  |  | (0.996, 1.036) |
| Maternal Postnatal Depression (14 months) |  |  |  | 1.013 |  | 1.003 |
|  |  |  |  | (0.994, 1.032) |  | (0.983, 1.023) |
| Maternal Postnatal Depression (3.5 Years) |  |  |  |  | 1.022^*^ | 1.014 |
|  |  |  |  |  | (1.002, 1.042) | (0.993, 1.036) |
| Maternal Depression (5 Years) | 1.024^***^ | 1.022^***^ | 1.019^***^ | 1.019^***^ | 1.017^**^ | 1.016^**^ |
|  | (1.015, 1.033) | (1.013, 1.031) | (1.009, 1.029) | (1.009, 1.029) | (1.007, 1.027) | (1.005, 1.026) |
| Note: Exp (ß) interpreted as geometric means. Maternal Age (Reference: 35+); Sex (Reference: female); Ethnicity (Reference: white British) *p<0.05; **p<0.01; ***p<0.001. Maternal Depression 5 Years Measured by CES-D. | | | | | | |

| **Child Mental Health Life Course Models (n=663)** | | | | | | |
| --- | --- | --- | --- | --- | --- | --- |
|  | Model I | Model II | Model III | Model IV | Model V | Model VI |
|  | Exp (ß) | Exp (ß) | Exp (ß) | Exp (ß) | Exp (ß) | Exp (ß) |
| **CBCL Internalising Raw Scores** |  |  |  |  |  |  |
| Household Income | 1.013 | 1.017 | 1.005 | 1.011 | 1.009 | 1.003 |
|  | (0.982, 1.045) | (0.980, 1.055) | (0.968, 1.042) | (0.975, 1.049) | (0.973, 1.046) | (0.967, 1.040) |
| Child Age |  | 0.975^**^ | 0.977^**^ | 0.975^**^ | 0.975^**^ | 0.976^**^ |
|  |  | (0.959, 0.992) | (0.960, 0.993) | (0.959, 0.992) | (0.959, 0.991) | (0.960, 0.992) |
| Maternal Age (18-24) |  | 1.024 | 0.982 | 1.029 | 1.005 | 0.984 |
|  |  | (0.820, 1.277) | (0.788, 1.224) | (0.825, 1.283) | (0.809, 1.250) | (0.791, 1.224) |
| Maternal Age (25-34) |  | 1.08 | 1.066 | 1.086 | 1.079 | 1.071 |
|  |  | (0.907, 1.286) | (0.897, 1.267) | (0.913, 1.293) | (0.909, 1.281) | (0.902, 1.271) |
| Sex (Male) |  | 1.029 | 1.024 | 1.024 | 1.035 | 1.031 |
|  |  | (0.913, 1.160) | (0.909, 1.153) | (0.909, 1.155) | (0.920, 1.165) | (0.916, 1.160) |
| Ethnicity (Other) |  | 1.277 | 1.263 | 1.283 | 1.335 | 1.315 |
|  |  | (0.884, 1.843) | (0.878, 1.816) | (0.890, 1.850) | (0.930, 1.916) | (0.917, 1.887) |
| Maternal Prenatal Depression |  |  | 1.032^***^ |  |  | 1.019^*^ |
|  |  |  | (1.016, 1.048) |  |  | (1.002, 1.037) |
| Maternal Postnatal Depression (14 months) |  |  |  | 1.020^*^ |  | 1.001 |
|  |  |  |  | (1.003, 1.037) |  | (0.983, 1.019) |
| Maternal Postnatal Depression (3.5 Years) |  |  |  |  | 1.045^***^ | 1.036^***^ |
|  |  |  |  |  | (1.027, 1.063) | (1.016, 1.056) |
| Maternal Depression (5 Years) | 1.028^***^ | 1.027^***^ | 1.022^***^ | 1.023^***^ | 1.017^***^ | 1.016^**^ |
|  | (1.019, 1.036) | (1.019, 1.035) | (1.014, 1.031) | (1.013, 1.032) | (1.008, 1.026) | (1.006, 1.025) |
| Note: Exp (ß) interpreted as geometric means. Maternal Age (Reference: 35+); Sex (Reference: female); Ethnicity (Reference: white British) *p<0.05; **p<0.01; ***p<0.001. Maternal Depression 5 Years Measured by CES-D. | | | | | | |
|  |  |  |  |  |  |  |

**S3: Multivariable Analysis CBCL Internalising Problems**

We found no significant association between household income at 20-weeks gestation and internalising problems at age 5 years. In sequential models, maternal mental health entered at any timepoint (prenatal, 14 months and 3.5 years postnatal) was associated with increased internalising scores. A one unit increase in EPDS score increased internalising scores by 4% (95% CI 2-6) in the prenatal period, by 4% (95% CI 2-5) at 14 months postnatal and 6% (95% CI 4-8) at 3.5 years. There was no significant association between child’s sex and internalising problem score.

| **Child Mental Health Life Course Models (n=664)** | |  |  |  |  |  |
| --- | --- | --- | --- | --- | --- | --- |
|  | Model I | Model II | Model III | Model IV | Model V | Model VI |
|  | Exp (ß) | Exp (ß) | Exp (ß) | Exp (ß) | Exp (ß) | Exp (ß) |
| **CBCL Internalising Raw Scores** |  |  |  |  |  |  |
| Household Income | 1.23 | 1.26 | 1.09 | 1.13 | 1.10 | 1.04 |
|  | (0.985, 1.535) | (0.968, 1.635) | (0.842, 1.421) | (0.869, 1.468) | (0.855, 1.422) | (0.803, 1.343) |
| Child Age |  | 0.971^***^ | 0.974^**^ | 0.972^**^ | 0.972^***^ | 0.973^**^ |
|  |  | (0.955, 0.988) | (0.957, 0.990) | (0.956, 0.989) | (0.956, 0.989) | (0.957, 0.990) |
| Maternal Age (18-24) |  | 1.017 | 0.963 | 1.029 | 0.996 | 0.978 |
|  |  | (0.810, 1.278) | (0.769, 1.205) | (0.822, 1.288) | (0.800, 1.241) | (0.785, 1.218) |
| Maternal Age (25-34) |  | 1.068 | 1.052 | 1.084 | 1.074 | 1.068 |
|  |  | (0.892, 1.279) | (0.882, 1.255) | (0.908, 1.295) | (0.903, 1.277) | (0.899, 1.270) |
| Sex (Male) |  | 1.058 | 1.044 | 1.039 | 1.05 | 1.041 |
|  |  | (0.935, 1.196) | (0.925, 1.178) | (0.920, 1.173) | (0.933, 1.182) | (0.925, 1.172) |
| Ethnicity (Other) |  | 1.211 | 1.208 | 1.242 | 1.32 | 1.3 |
|  |  | (0.830, 1.768) | (0.834, 1.749) | (0.856, 1.803) | (0.917, 1.901) | (0.904, 1.870) |
| Maternal Prenatal Depression |  |  | 1.044^***^ |  |  | 1.021^*^ |
|  |  |  | (1.028, 1.061) |  |  | (1.003, 1.039) |
| Maternal Postnatal Depression (14 months) |  |  |  | 1.038^***^ |  | 1.008 |
|  |  |  |  | (1.022, 1.054) |  | (0.991, 1.026) |
| Maternal Postnatal Depression (3.5 Years) |  |  |  |  | 1.060^***^ | 1.046^***^ |
|  |  |  |  |  | (1.044, 1.076) | (1.027, 1.066) |
| Note: Exp (ß) interpreted as geometric means. Maternal Age (Reference: 35+); Sex (Ref: female); Ethnicity (Reference: white British) *p<0.05; **p<0.01; ***p<0.001. Household Income represents the socioeconomic (SEC) gap, the difference between the most and least deprived households. | | | | | | |
|  |  |  |  |  |  |  |

**S4: Sensitivity analysis using the English Index of Multiple Deprivation (IMD) as an alternative measure of socioeconomic conditions (SECs).**

| **Sensitivity: Child Mental Health Life Course Models (n=663)** | | | | | | |
| --- | --- | --- | --- | --- | --- | --- |
|  | Model I | Model II | Model III | Model IV | Model V | Model VI |
|  | Exp (ß) | Exp (ß) | Exp (ß) | Exp (ß) | Exp (ß) | Exp (ß) |
| **CBCL Externalising Raw Scores** |  |  |  |  |  |  |
| IMD 1 | 1.394^*^ | 1.372^*^ | 1.342^*^ | 1.323^*^ | 1.383^*^ | 1.345^*^ |
|  | (1.081, 1.797) | (1.061, 1.775) | (1.040, 1.731) | (1.024, 1.708) | (1.074, 1.781) | (1.044, 1.733) |
| IMD 2 | 1.249 | 1.239 | 1.228 | 1.192 | 1.255 | 1.226 |
|  | (0.949, 1.644) | (0.942, 1.631) | (0.936, 1.610) | (0.907, 1.565) | (0.958, 1.644) | (0.936, 1.607) |
| IMD 3 | 1.400^*^ | 1.377^*^ | 1.362^*^ | 1.349^*^ | 1.408^**^ | 1.379^*^ |
|  | (1.079, 1.815) | (1.060, 1.788) | (1.052, 1.763) | (1.041, 1.748) | (1.089, 1.821) | (1.067, 1.784) |
| IMD 4 | 1.432^*^ | 1.391^*^ | 1.414^*^ | 1.374^*^ | 1.455^*^ | 1.438^*^ |
|  | (1.045, 1.963) | (1.015, 1.907) | (1.036, 1.931) | (1.005, 1.877) | (1.067, 1.985) | (1.055, 1.961) |
| Maternal Age (18-24) |  | 1.048 | 0.964 | 1.027 | 0.998 | 0.965 |
|  |  | (0.833, 1.319) | (0.765, 1.214) | (0.818, 1.290) | (0.795, 1.252) | (0.767, 1.213) |
| Maternal Age (25-34) |  | 1.047 | 1.03 | 1.06 | 1.046 | 1.042 |
|  |  | (0.858, 1.278) | (0.846, 1.254) | (0.870, 1.291) | (0.860, 1.272) | (0.857, 1.267) |
| Sex (Male) |  | 1.203^**^ | 1.193^*^ | 1.187^*^ | 1.198^**^ | 1.189^*^ |
|  |  | (1.050, 1.377) | (1.043, 1.363) | (1.038, 1.357) | (1.049, 1.369) | (1.041, 1.358) |
| Ethnicity (Other) |  | 0.835 | 0.827 | 0.849 | 0.885 | 0.868 |
|  |  | (0.550, 1.266) | (0.548, 1.248) | (0.562, 1.283) | (0.587, 1.334) | (0.577, 1.307) |
| Maternal Prenatal Depression |  |  | 1.037^***^ |  |  | 1.019 |
|  |  |  | (1.019, 1.054) |  |  | (0.999, 1.039) |
| Maternal Postnatal Depression (14 months) |  |  |  | 1.033^***^ |  | 1.012 |
|  |  |  |  | (1.016, 1.051) |  | (0.992, 1.032) |
| Maternal Postnatal Depression (3.5 Years) |  |  |  |  | 1.043^***^ | 1.028^**^ |
|  |  |  |  |  | (1.025, 1.062) | (1.007, 1.050) |
|  | | | | | | |

Note: Exp (ß) interpreted as geometric means. IMD: English Index of Multiple Deprivation (Reference: IMD 5 Least Deprived Quintile); Maternal Age (Reference: 35+); Sex (Ref: female); Ethnicity (Reference: white British) *p<0.05; **p<0.01; ***p<0.001.

| **Sensitivity: Child Mental Health Life Course Models (n=663)** | | | | | | | |
| --- | --- | --- | --- | --- | --- | --- | --- |
|  | Model I | Model II | Model III | Model IV | Model V | Model VI |  |
|  | Exp (ß) | Exp (ß) | Exp (ß) | Exp (ß) | Exp (ß) | Exp (ß) |  |
| **CBCL Internalising Raw Scores** |  |  |  |  |  |  |  |
| IMD 1 | 1.227 | 1.21 | 1.176 | 1.156 | 1.223 | 1.191 |  |
|  | (0.973, 1.547) | (0.955, 1.532) | (0.934, 1.481) | (0.916, 1.459) | (0.975, 1.534) | (0.950, 1.494) |  |
| IMD 2 | 1.093 | 1.082 | 1.07 | 1.031 | 1.102 | 1.079 |  |
|  | (0.851, 1.404) | (0.841, 1.393) | (0.836, 1.368) | (0.805, 1.322) | (0.866, 1.403) | (0.848, 1.374) |  |
| IMD 3 | 1.188 | 1.176 | 1.161 | 1.147 | 1.215 | 1.191 |  |
|  | (0.937, 1.506) | (0.925, 1.495) | (0.918, 1.467) | (0.906, 1.452) | (0.965, 1.530) | (0.947, 1.499) |  |
| IMD 4 | 1.174 | 1.166 | 1.19 | 1.147 | 1.243 | 1.231 |  |
|  | (0.880, 1.565) | (0.873, 1.557) | (0.897, 1.578) | (0.864, 1.524) | (0.941, 1.640) | (0.933, 1.623) |  |
| Maternal Age (18-24) |  | 1.064 | 0.958 | 1.039 | 0.992 | 0.956 |  |
|  |  | (0.862, 1.315) | (0.777, 1.181) | (0.844, 1.278) | (0.810, 1.215) | (0.779, 1.172) |  |
| Maternal Age (25-34) |  | 1.068 | 1.046 | 1.084 | 1.067 | 1.061 |  |
|  |  | (0.890, 1.283) | (0.875, 1.251) | (0.906, 1.297) | (0.895, 1.271) | (0.891, 1.263) |  |
| Sex (Male) |  | 1.038 | 1.027 | 1.021 | 1.032 | 1.024 |  |
|  |  | (0.916, 1.175) | (0.909, 1.159) | (0.903, 1.153) | (0.916, 1.163) | (0.910, 1.154) |  |
| Ethnicity (Other) |  | 1.234 | 1.22 | 1.261 | 1.342 | 1.315 |  |
|  |  | (0.842, 1.809) | (0.840, 1.771) | (0.866, 1.835) | (0.930, 1.937) | (0.912, 1.895) |  |
| Maternal Prenatal Depression |  |  | 1.046^***^ |  |  | 1.021^*^ |  |
|  |  |  | (1.031, 1.063) |  |  | (1.003, 1.039) |  |
| Maternal Postnatal Depression (14 months) |  |  |  | 1.041^***^ |  | 1.01 |  |
|  |  |  |  | (1.025, 1.057) |  | (0.992, 1.028) |  |
| Maternal Postnatal Depression (3.5 Years) |  |  |  |  | 1.063^***^ | 1.047^***^ |  |
|  |  |  |  |  | (1.046, 1.079) | (1.028, 1.067) |  |
|  | | | | | | | |

Note: Exp (ß) interpreted as geometric means. IMD: English Index of Multiple Deprivation (Reference: IMD 5 Least Deprived Quintile); Maternal Age (Reference: 35+); Sex (Ref: female); Ethnicity (Reference: white British) *p<0.05; **p<0.01; ***p<0.001.

**S5: Sensitivity analysis using clinical cut-offs on the child behaviour checklist (CBCL) as an alternative outcome measure.**

We repeated our hierarchical approach to multivariate regression model using clinical cut-offs as an alternative outcome. We dichotomised CBCL T-score (age and sex standardised total score) for internalising and externalising problems using established cut-offs. [Reference 0: no clinical-level scores; 1: borderline-clinical scores] [1]

[1] Achenbach, T. M. (1991). Manual for the child behavior checklist/4-18 and 1991 profile. Burlington VT.enbach TM. Manual for the child behavior checklist/4-18 and 1991 profile. *Burlingt VT* 1991.

| **Sensitivity: Child Mental Health Life Course Models (n=665)** | | | | | | |
| --- | --- | --- | --- | --- | --- | --- |
|  | Model I | Model II | Model III | Model IV | Model V | Model VI |
|  | Exp (ß) | Exp (ß) | Exp (ß) | Exp (ß) | Exp (ß) | Exp (ß) |
| **CBCL Clinical Internalising Scores** | | | | | | |
| Household Income | 1.156^*^ | 1.182 | 1.095 | 1.134 | 1.098 | 1.062 |
|  | (1.004, 1.337) | (0.999, 1.406) | (0.920, 1.308) | (0.955, 1.352) | (0.924, 1.310) | (0.890, 1.272) |
| Maternal Age (18-24) |  | 1.081 | 0.876 | 1.117 | 0.913 | 0.808 |
|  |  | (0.390, 3.345) | (0.307, 2.759) | (0.400, 3.475) | (0.315, 2.907) | (0.275, 2.601) |
| Maternal Age (25-34) |  | 1.39 | 1.326 | 1.46 | 1.48 | 1.417 |
|  |  | (0.605, 3.773) | (0.567, 3.649) | (0.632, 3.980) | (0.632, 4.079) | (0.601, 3.923) |
| Sex (Male) |  | 0.976 | 0.958 | 0.918 | 0.923 | 0.947 |
|  |  | (0.563, 1.683) | (0.546, 1.672) | (0.525, 1.593) | (0.522, 1.624) | (0.533, 1.675) |
| Ethnicity (Other) |  | 0.567 | 0.557 | 0.575 | 0.778 | 0.767 |
|  |  | (0.031, 2.879) | (0.030, 2.939) | (0.031, 2.994) | (0.042, 4.093) | (0.041, 4.050) |
| Maternal Prenatal Depression |  |  | 1.157^***^ |  |  | 1.100^*^ |
|  |  |  | (1.086, 1.235) |  |  | (1.021, 1.183) |
| Maternal Postnatal Depression (14 months) |  |  |  | 1.089^**^ |  | 0.989 |
|  |  |  |  | (1.023, 1.158) |  | (0.916, 1.064) |
| Maternal Postnatal Depression (3.5 Years) |  |  |  |  | 1.193^***^ | 1.159^***^ |
|  |  |  |  |  | (1.119, 1.274) | (1.077, 1.250) |
|  | | | | | | |

Note: Exp (ß) interpreted as geometric means. Maternal Age (Reference: 35+); Sex (Ref: female); Ethnicity (Reference: white British) *p<0.05; **p<0.01; ***p<0.001. Household Income represents the socioeconomic (SEC) gap, the difference between the most and least deprived households

| **Sensitivity: Child Mental Health Life Course Models (n=665)** | | | | | | |
| --- | --- | --- | --- | --- | --- | --- |
|  | Model I | Model II | Model III | Model IV | Model V | Model VI |
|  | Exp (ß) | Exp (ß) | Exp (ß) | Exp (ß) | Exp (ß) | Exp (ß) |
| **CBCL Clinical Externalising Scores** |  |  |  |  |  |  |
| Household Income | 1.241^*^ | 1.340^*^ | 1.279 | 1.307^*^ | 1.26 | 1.244 |
|  | (1.018, 1.529) | (1.056, 1.721) | (1.004, 1.647) | (1.027, 1.683) | (0.990, 1.621) | (0.975, 1.605) |
| Maternal Age (18-24) |  | 0.563 | 0.501 | 0.567 | 0.472 | 0.45 |
|  |  | (0.152, 2.386) | (0.134, 2.141) | (0.153, 2.411) | (0.121, 2.056) | (0.115, 1.971) |
| Maternal Age (25-34) |  | 0.887 | 0.864 | 0.902 | 0.905 | 0.893 |
|  |  | (0.315, 3.167) | (0.304, 3.101) | (0.319, 3.224) | (0.318, 3.254) | (0.313, 3.217) |
| Sex (Male) |  | 1.728 | 1.726 | 1.681 | 1.695 | 1.72 |
|  |  | (0.811, 3.803) | (0.805, 3.820) | (0.784, 3.714) | (0.785, 3.775) | (0.795, 3.842) |
| Ethnicity (Other) |  | 0 | 0 | 0 | 0 | 0 |
|  |  |  |  |  |  |  |
| Maternal Prenatal Depression |  |  | 1.091 |  |  | 1.043 |
|  |  |  | (0.997, 1.189) |  |  | (0.940, 1.152) |
| Maternal Postnatal Depression (14 months) |  |  |  | 1.055 |  | 0.99 |
|  |  |  |  | (0.967, 1.143) |  | (0.894, 1.086) |
| Maternal Postnatal Depression (3.5 Years) |  |  |  |  | 1.137^**^ | 1.125^*^ |
|  |  |  |  |  | (1.047, 1.235) | (1.023, 1.235) |
|  | | | | | | |

Note: Exp (ß) interpreted as geometric means. Maternal Age (Reference: 35+); Sex (Ref: female); Ethnicity (Reference: white British) *p<0.05; **p<0.01; ***p<0.001. Household Income represents the socioeconomic (SEC) gap, the difference between the most and least deprived households

**S6: Untransformed CBCL Externalising Scores**

We repeated our hierarchical approach to multivariate regression model using untransformed CBCL raw scores. The conclusions are similar to our main analysis. Using the untransformed CBCL scores, the income gap shows a 2.8-point difference between the least and most deprived children in externalising problems in model VI.

| **Sensitivity: Untransformed CBCL Scores** | | |  |  |  |  |
| --- | --- | --- | --- | --- | --- | --- |
|  | Model I | Model II | Model III | Model IV | Model V | Model VI |
| **CBCL Externalising Raw Scores** |  | | | | | |
| Household Income | 0.428^**^ | 0.545^***^ | 0.427^**^ | 0.471^**^ | 0.443^**^ | 0.395^*^ |
|  | (0.163, 0.694) | (0.233, 0.856) | (0.113, 0.741) | (0.156, 0.785) | (0.134, 0.753) | (0.081, 0.708) |
| Child Age |  | -0.227^**^ | -0.212^**^ | -0.222^**^ | -0.221^**^ | -0.214^**^ |
|  |  | (-0.370, -0.083) | (-0.354, -0.070) | (-0.365, -0.079) | (-0.362, -0.080) | (-0.355, -0.072) |
| Maternal Age (18-24) |  | -0.857 | -1.183 | -0.803 | -0.968 | -1.128 |
|  |  | (-2.754, 1.039) | (-3.067, 0.702) | (-2.691, 1.086) | (-2.836, 0.900) | (-3.003, 0.747) |
| Maternal Age (25-34) |  | -0.166 | -0.257 | -0.095 | -0.139 | -0.194 |
|  |  | (-1.661, 1.329) | (-1.738, 1.223) | (-1.584, 1.393) | (-1.611, 1.333) | (-1.666, 1.278) |
| Sex (Male) |  | 1.754^***^ | 1.675^**^ | 1.667^**^ | 1.716^***^ | 1.676^**^ |
|  |  | (0.730, 2.777) | (0.661, 2.689) | (0.646, 2.687) | (0.708, 2.724) | (0.667, 2.684) |
| Ethnicity (Other) |  | -1.953 | -1.972 | -1.833 | -1.49 | -1.598 |
|  |  | (-5.097, 1.191) | (-5.083, 1.140) | (-4.964, 1.298) | (-4.592, 1.611) | (-4.697, 1.500) |
| Maternal Prenatal Depression |  |  | 0.256^***^ |  |  | 0.147 |
|  |  |  | (0.126, 0.387) |  |  | (-0.002, 0.296) |
| Maternal Postnatal Depression (14 months) |  |  |  | 0.179^**^ |  | 0.008 |
|  |  |  |  | (0.048, 0.309) |  | (-0.143, 0.158) |
| Maternal Postnatal Depression (3.5 Years) |  |  |  |  | 0.313^***^ | 0.244^**^ |
|  |  |  |  |  | (0.182, 0.445) | (0.087, 0.401) |

Note: Maternal Age (Reference: 35+); Sex (Ref: female); Ethnicity (Reference: white British) *p<0.05; **p<0.01; ***p<0.001.

**S7: Clinical Cut-offs EPDS**

We repeated our main analysis using a validated cut-off for the EPDS measure**.** We did not include this in our main analysis due to the small sample size, and because dichotomising the EPDS score means that we lose information by only focus on the mediating role of the most severe end of the maternal mental health spectrum.

We also undertook a formal mediation analysis using the counterfactual framework to assess how much of the effect of SEC income on child mental health is mediated via maternal mental health (dichotomised as a clinical cut-off) measured at all three time points. This process is described in S1.

Overall 8.8 % of the total effect of socio-economic conditions (low versus high) on child mental health is mediated through maternal mental health with a total effect of 1.05 (95% CI 1.013 to 1.098) and NIE of 1.01 (95%CI 0.998 to 1.012).

|  | Model I | Model II | Model III | Model IV | Model V | Model VI |
| --- | --- | --- | --- | --- | --- | --- |
|  | Exp (ß) | Exp (ß) | Exp (ß) | Exp (ß) | Exp (ß) | Exp (ß) |
| **CBCL Externalising Raw Scores** |  | | | | | |
| Household Income | 1.041^*^ | 1.055^*^ | 1.051^*^ | 1.054^*^ | 1.053^*^ | 1.050^*^ |
|  | (1.005, 1.078) | (1.012, 1.099) | (1.009, 1.095) | (1.011, 1.098) | (1.011, 1.097) | (1.007, 1.094) |
| Child Age |  | 0.972^**^ | 0.973^**^ | 0.972^**^ | 0.972^**^ | 0.973^**^ |
|  |  | (0.953, 0.990) | (0.954, 0.991) | (0.954, 0.990) | (0.954, 0.990) | (0.955, 0.991) |
| Maternal Age (18-24) |  | 0.954 | 0.938 | 0.954 | 0.951 | 0.936 |
|  |  | (0.744, 1.224) | (0.731, 1.203) | (0.743, 1.224) | (0.741, 1.221) | (0.730, 1.202) |
| Maternal Age (25-34) |  | 1.05 | 1.046 | 1.05 | 1.052 | 1.047 |
|  |  | (0.863, 1.278) | (0.859, 1.272) | (0.863, 1.278) | (0.864, 1.281) | (0.861, 1.275) |
| Sex (Male) |  | 1.240^**^ | 1.248^**^ | 1.238^**^ | 1.240^**^ | 1.248^**^ |
|  |  | (1.084, 1.418) | (1.091, 1.428) | (1.082, 1.417) | (1.084, 1.418) | (1.090, 1.428) |
| Ethnicity (Other) |  | 0.802 | 0.806 | 0.802 | 0.807 | 0.809 |
|  |  | (0.531, 1.213) | (0.534, 1.217) | (0.531, 1.213) | (0.533, 1.220) | (0.535, 1.222) |
| Maternal Prenatal Depression |  |  | 1.285 |  |  | 1.272 |
|  |  |  | (1.000, 1.651) |  |  | (0.986, 1.642) |
| Maternal Postnatal Depression (14 months) |  |  |  | 1.069 |  | 1.013 |
|  |  |  |  | (0.790, 1.448) |  | (0.741, 1.383) |
| Maternal Postnatal Depression (3.5 Years) |  |  |  |  | 1.129 | 1.076 |
|  |  |  |  |  | (0.818, 1.558) | (0.772, 1.500) |

Note: Maternal Age (Reference: 35+); Sex (Ref: female); Ethnicity (Reference: white British) *p<0.05; **p<0.01; ***p<0.001.

| **Effect Breakdown** | **(ß)** | **95% LCL** | **95% UCL** | **Exp (ß)** | **95% LCL** | **95% UCL** |
| --- | --- | --- | --- | --- | --- | --- |
| Natural Direct Effect | 0.049 | 0.008 | 0.089 | 1.050 | 1.008 | 1.093 |
| Natural Indirect Effect | 0.005 | -0.002 | 0.012 | 1.005 | 0.998 | 1.012 |
| Total Effect | 0.053 | 0.013 | 0.093 | 1.055 | 1.013 | 1.098 |
| Exposure: household Income. Confounders: maternal age, ethnicity, child sex, maternal mental health | | | | | | |
| (20 weeks gestation, 14 months & 3.5 years postnatal) | | |  |  |  |  |

**Mediation Analysis Results**

**S8: Table showing comparison of baseline and analysis sample demographics.**

| **Table: Comparison of sample demographics** |  |  |  |
| --- | --- | --- | --- |
|  | **Baseline Sample** | **Analysis Sample** | |
| **Subjects n** | 1233 | 666 | |
| **Externalising raw score** | 8.4 (7.7) | 7.7 (6.8) | |
| **Internalising raw score** | 6.7 (6.3) | 6.2 (5.5) | |
| **Log-Externalising raw score** | 1.9 (0.9) | 1.8 (0.9) | |
| **Log-Internalising raw score** | 1.7 (0.8) | 1.7 (0.8) | |
| **Income Band (%)** |  |  | |
| Up to £10,000 | 104 (10.2) | 48 (7.2) | |
| £10-20,000 | 137 (13.4) | 77 (11.6) | |
| £21-30,000 | 162 (15.9) | 97 (14.6) | |
| £31-40,000 | 195 (19.1) | 134 (20.1) | |
| £41-50,000 | 163 (16.0) | 110 (16.5) | |
| £51-60,000 | 130 (12.7) | 101 (15.2) | |
| £61-70,000 | 61 (6.0) | 46 (6.9) | |
| Over £71,000 | 68 (6.7) | 53 (8.0) | |
| **Maternal Age (%)** |  |  | |
| 18-24 | 489 (39.8) | 160 (24.1) | |
| 25-34 | 607 (49.3) | 408 (61.4) | |
| 35+ | 134 (10.9) | 96 (14.5) | |
| **Ethnicity (%)** |  |  | |
| Other | 45 (3.6) | 19 (2.9) | |
| **Sex (%)** |  |  | |
| Male | 599 (48.6) | 317 (47.6) | |
| **IMD Quintile** |  |  | |
| 1 (Most Deprived) | 514 (41.8) | 229 (34.5) | |
| 2 | 225 (18.3) | 126 (19.0) | |
| 3 | 294 (23.9) | 186 (28.1) | |
| 4 | 105 (8.5) | 63 (9.5) | |
| 5 (Least Deprived) | 92 (7.5) | 59 (8.9) | |
| **Maternal Mental Health** |  |  | |
| Prenatal | 6.8 (4.1) | 6.4 (4.0) | |
| Postnatal (14 months) | 5.2 (3.6) | 5.1 (4.0) | |
| Postnatal (3.5 years) | 5.3 (3.4) | 5.0 (3.9) | |
| Note: Analysis sample: Participants whom had exposure and outcome data available | | |  |

**S9: Flowchart of Wirral Child Health & Development Study (WCHADS) analysis sample**

664 participants entered into analysis

759 participants with mental health data

1233 participants available for post-natal follow-up

Removing missing data for analysis

- Excluded if missing on any variables in analysis: Total missing (n=95) This was made up of a combination of missingness on the following variables: Household income at 20-weeks gestation (n=93); maternal age at recruitment to study (n=2).
- 474 participants did not have data on the Child Behaviour Checklist (CBCL) at the outcome timepoint (child aged 5 years, wave 12)

Applying inclusion/exclusion criteria for study
